# Irido-Choroiditis Following Meningitis

**Published:** 1885-06

**Authors:** A. W. Calhoun

**Affiliations:** Professor of Diseases of the Eye, Ear and Throat, in the Atlanta Medical College, Atlanta, Georgia


### Clinic Reports.

### IRIDO-CHOROIDITIS FOLLOWING MENINGITIS.

A CLINICAL LECTURE BY A. W. CALHOUN, M. D., PROFESSOR OF DISEASES OF THE EYE, EAR AND THROAT, IN THE ATLANTA MEDICAL COLLEGE, ATLANTA, GEORGIA.

Reported for the ATLANTA MEDICAL AND SURGICAL JOURNAL.

Of all the sequelæ of meningitis this, so far as my observation extends, is the rarest, only five cases having occurred in my practice.

In each case the patient recovered from the meningitis. The disease of the eye begins during the violence of the inflammation of the brain, beginning apparently as aniritis, but upon more thorough examination it will be found that it had already begun in the choroid and extended to the iris, the iris being secondarily involved.

The vitreous humor is found filled with an effusion of lymph to the extent that, looking through the pupil into the interior of the ball, it presents the appearance of being filled with pus. In all of these cases the iris has become firmly adherent to the lens, being entirely uninfluenced by strong solutions of atropia.

There is no great pain at any time during the disease, nor would the redness of the exterior portions of the eye indicate anything very serious, but upon testing the vision you will find complete blindness in every case.

The very nature of the case precludes the possibility of any restoration of sight. The abundant effusion in the cavity of the ball, causing complete destruction of the retinal tissue.

In each of the cases under my observation, the ages ranged from two to twelve years, and, curiously, in four of the cases it occurred in the left eye. I regard this, however, as a mere coincidence, as there can be no reason why it should more readily occur upon one side than the other.

The balls usually shrink slightly, but not enough to cause great deformity.

In every one of these cases the cornea has remained intact.

### PATHOLOGY OF THE DISEASE.

The inflammation of the eye is set up evidently by an extension of the inflammation of the meninges of the brain along the sheath of the optic nerve through the optic foramen to the interior of the ball. The optic sheath is nothing but a continuation of the dura mater; hence, you can readily see how easy it would be for inflammation to extend from the brain to the ball.

### TREATMENT.

The treatment practically amounts to nothing. The patient is irretrievably blinded, and all we can do is to use atropia, with a view to reducing as much iritic inflammation as possible. Counter-irritation may sometimes be advantageously employed in connection with the atropia; and in case more than ordinary pain exist, opiates would be indicated.

During the acute inflammatory stage, and also during the process of shrinking of the ball, there is considerable sympathetic irritation in the sound eye which disappears upon the relief of the inflammation in the diseased eye.

In one of the cases a complication arose which may ultimately necessitate an operation. The anterior chamber suddenly filled with pus, coming from the rear of the lens. In its present position it acts decidedly as a foreign body and will sooner or later bring about more or less violent inflammation, requiring an operation for its removal, if not soon absorbed.

Atrophy of the optic nerve is a frequent result of meningitis, but irido-choroiditis, with this large amount of effusion of lymph in the vitreous humor, is so rare as to confine my experience with it to these five cases.

